# Urinary Neutrophil Gelatinase-Associated Lipocalin predicts the severity of contrast-induced acute kidney injury in chronic kidney disease patients undergoing elective coronary procedures

**DOI:** 10.1186/1471-2369-14-270

**Published:** 2013-12-05

**Authors:** Adis Tasanarong, Pisit Hutayanon, Dilok Piyayotai

**Affiliations:** 1 Nephrology Unit, Department of Medicine, Faculty of Medicine, Thammasat University (Rangsit Campus), Khlong Nueng, Khlong Luang, Pathum Thani 12121, Thailand; 2 Cardiology Unit, Department of Medicine, Faculty of Medicine, Thammasat University (Rangsit Campus), Khlong Nueng, Khlong Luang, Pathum Thani 12121, Thailand

**Keywords:** Neutrophil Gelatinase-Associated Lipocalin (NGAL), Contrast Induced Acute Kidney Injury (CI-AKI), Chronic Kidney injury (CKD), Coronary procedures

## Abstract

**Background:**

Contrast-induced acute kidney injury (CI-AKI) particularly in high risk patients with chronic kidney disease (CKD), increases morbidity and mortality. Neutrophil gelatinase-associated lipocalin (NGAL) is a protein excreted by the kidney during AKI. There are no urine (u) NGAL data as an early CI-AKI marker in CKD patients undergoing coronary procedures.

**Methods:**

This prospective study enrolled 130 patients with estimated glomerular filtration rate (eGFR) < 60 ml/min/1.73 m^2^ undergoing elective coronary procedures. Serial urine samples, obtained at baseline and 3, 6, 12, 18, and 24 h post contrast administration were analyzed by NGAL ELISA kit. AKI was defined as an increase in serum creatinine (SCr) of ≥ 0.3 mg/dl or ≥ 1.5 times baseline SCr within 48 h per 2012 KDIGO guidelines. Receiver operator characteristic curve analyses identified optimal uNGAL and delta of uNGAL values for diagnosing CI-AKI.

**Results:**

The uNGAL was significantly and inverse correlated with eGFR (*R =* 0.25, P < 0.005). CI-AKI developed in 16/130 (12.31%) patients: 13 and 3 in CI-AKI stages I and II, respectively. uNGAL and delta of uNGAL were significantly higher in the CI-AKI group when compared with the No CI-AKI group (P < 0.05). The best uNGAL cut-off for optimal sensitivity 94%, specificity 78%, and area under the curve 0.84 for predicting CI-AKI was 117 ng/mL at 6 h, respectively. Corresponding values for predicting CI-AKI stage II were 100%, 87% and 0.9 when using an uNGAL of 264 ng/mL at 6 h.

**Conclusions:**

Monitoring of uNGAL levels not only provide the early detecting CI-AKI but also predict the severity of CI-AKI in CKD patients undergoing elective coronary procedures.

## Background

Contrast-induced acute kidney injury (CI-AKI) is one of the most common complications in patients who receive intravenous contrast media [1-4]. The incidence of CI-AKI is low 1–2% in patients with normal renal function even with underlying diabetes mellitus [1] but increases up to 25% in patients with certified risk factors, for instance the combination of chronic kidney disease (CKD) and diabetes, congestive heart failure, advanced age, and concurrent use of nephrotoxic drugs [4] with raising the morbidity and mortality [5]. CI-AKI requiring dialysis occurs in 3-4% of patients with underlying CKD undergoing coronary procedures [6,7] and CI-AKI in CKD patients contributes to extended hospitalizations and increases long-term morbidity and mortality [8].

CI-AKI is defined as an acute deterioration in renal function after intravenous administration of contrast media by an absolute increase in serum creatinine (SCr) ≥ 0.5 mg/dL or ≥ 25% relative increase from baseline SCr within 48 hours after contrast media injection without evidence of other causes. In general, impairment of renal function in CI-AKI occurs within 3 days after intravenous administration of contrast media, while the peak of SCr is observed at 3–5 days and returns to the baseline value within 1–3 weeks [9,10]. However, SCr is an unreliable biomarker during AKI in kidney function [11,12] because many factors can affect SCr concentration, including creatinine generation by muscle catabolism, diet, age, hydration status and renal tubular secretion of creatinine. In addition, during an acute decrease in glomerular filtration rate, SCr does not accurately represent kidney function in AKI until steady state has been reached, which requires several days [13,14]. Consequently, a changing of SCr within 48 hours after contrast media injection might result in a delay for diagnosis CI-AKI. The earlier detection of CI-AKI with another biomarker/s could be diagnostically and therapeutically beneficial.

The Neutrophil Gelatinase-Associated Lipocalin (NGAL) is a protein covalently bound to gelatinase in neutrophils which is usually expressed at very low concentrations in several human tissues, including kidney, lung, stomach, and colon [15]. During AKI, NGAL expression is markedly induced systemically and in injured distal nephron epithelium. Urine (u) NGAL concentrations are increased because of impaired reabsorption of NGAL by the damaged proximal tubules and direct excretion by the damaged distal tubules [16]. NGAL was detected easily in the blood and urine soon after AKI in experimental and clinical studies [17,18]. Therefore, NGAL has proved an early, sensitive, non-invasive biomarker for AKI in different clinical settings such as in cardiac surgery [19,20], critical care [21,22], and kidney transplantation [23,24].

Previous studies of uNGAL in CI-AKI have been conducted in adult and pediatric patients with normal renal function [25-28]. Given the lack of data in CKD patients undergoing coronary procedures, the present study was conducted to evaluate uNGAL as an early biomarker for the diagnosis and severity of CI-AKI.

## Methods

### Patient population

The prospective cohort study was conducted in patients who underwent clinically-driven, elective coronary angiography and/or intervention at Thammasat Chalerm Prakiat Hospital during the period from January 2010 to December 2011. Approval was obtained from the Ethics Committee of the Faculty of Medicine, Thammasat University. All patients provided written, informed consent to participate in the study. Patients aged ≥ 18 years with baseline estimation of glomerular filtration rate (eGFR) < 60 mL/min, as measured in their most recent sample and at least two measurements for ≥ three months prior to the beginning of the protocol, were included in the study. Stages of CKD were classified by the National Kidney Foundation practice guidelines [29]. However, patients who younger than 18 years, with pre-existing AKI, CKD stage 5 or unstable renal function (as evidenced by a change in SCr of ≥ 0.5 mg/dL, or ≥ 25%, within 14 days prior to the study) were excluded from further participation. Subjects were also excluded if they had a known allergy to any of the contrast agents, or were receiving mechanical ventilation, or suffered from congestive heart failure, cardiogenic shock or emergent angiography. Moreover, those receiving NAC, mannitol, diuretics, theophylline, dopamine, and ascorbic acid or contrast agents within 14 days before study commencement were not included.

All subjects were maintained in a euvolemic state with intravenous isotonic (0.9%) saline at rate 1 mL/kg per hour for 12 hours before and 12 hours after elective coronary procedures. Variation in the hydration rate was allowed for adjustments according to the clinical heart failure of individual patient. Hospital procedures mandated accurate hourly recording of all in-hospital volume inputs for patients undergoing elective coronary interventions. All patients were encouraged to drink if they were thirsty. Coronary intervention was performed using a standard protocol, via either the radial or femoral approach by the attending interventional cardiologist. All procedures were performed using low-osmolar, nonionic contrast media agent (Iopromide, Schering AG, Germany) in doses adjusted for body weight and determined by the number and location of cardiovascular angiograms.

### Blood and urine sample collections and storage

Venous blood samples were collected for the measurement of complete blood count, and SCr baseline levels 12–24 hrs prior to intervention and again 48 hrs after the procedure. eGFR was calculated by using the reexpressed 4-variable Modification of Diet in Renal Disease (MDRD) equation, where eGFR = 175 × plasma creatinine^-1.154^ × age^-0.203^ (× 0.742 if female; × 1.212 if African American) [30]. Serial urine samples were collected at baseline (prior to coronary procedures), and at 3, 6, 12, 18 and 24 h post coronary procedures. Samples were centrifuged at 2,000 g for 5 min and the supernatants stored at -70°C until assayed.

uNGAL concentrations were measured using a commercially available ELISA kit (Antibody Shop, Gentofte, Denmark), following the manufacturer’s instructions. All urine specimens were diluted to achieve concentration for optimal density according to the ELISA kit instruction before performing an ELISA assay to fit the concentrations of respective NGAL protein in the linear range of the standard curve. The inter-assay and intra-assay coefficients of variation for NGAL were < 5%. The measurements were made in duplicate and in a blinded fashion. NGAL levels were expressed in units of nanograms per milliliter.

Newer criteria of AKI from KDIGO guideline 2012 were applied for the diagnosis of CI-AKI. AKI was defined as an increase in SCr of ≥ 0.3 mg/dl or ≥ 1.5 times baseline creatinine within 48 hr, and the patients were classified into three stages according to the SCr criteria. Stage I defined as a SCr increase of ≥ 0.3 mg/dl or ≥ 1.5 to 1.9 times baseline; stage II as a SCr increase to ≥ 2.0 to 2.9 times and stage III of more than 3 times baseline. Urine criteria of AKI were not utilized in this study because of potential changes in urinary volume induced by some medical therapy.

The risk score for predicting CI-AKI development was calculated according to Mehran et al. [31]. Risk score assessment was evaluated by definition of clinical and cut-off point of laboratory investigation. Specific clinical and laboratory data were obtained from the hospital charts that were reviewed by research investigators.

### Statistical analysis

Values are presented as mean ± standard error of the mean (SE). A two-sample t-test or the non-parametric Mann–Whitney U test was used to compare continuous variables. Categorical variables were compared using the chi-square test or Fisher’s exact test, as appropriate. The associations between two continuous variables were assessed by Pearson’s correlation coefficient; all non-normally distributed values were log-transformed as required. To measure the sensitivity and specificity of uNGAL for the diagnosis of CI-AKI and its severity, receiver-operating characteristic (ROC) curves were generated and the area under the curve (AUC) calculated. We defined the cut-off as closest point to sensitivity = specificity = 1.0 on ROC curve. An AUC-ROC value of 0.90 to 1.0 indicated excellent, 0.80 to 0.89 good, 0.70 to 0.79 fair, 0.60 to 0.69 poor, and 0.50 to 0.59 indicated no useful value. The AUC ROCs were compared according to the method of Hanley and McNeil [32]. Two tailed p < 0.05 was considered statistically significant. The SPSS version 16 for Windows software was used for analyses.

## Results

**Figure 1:**
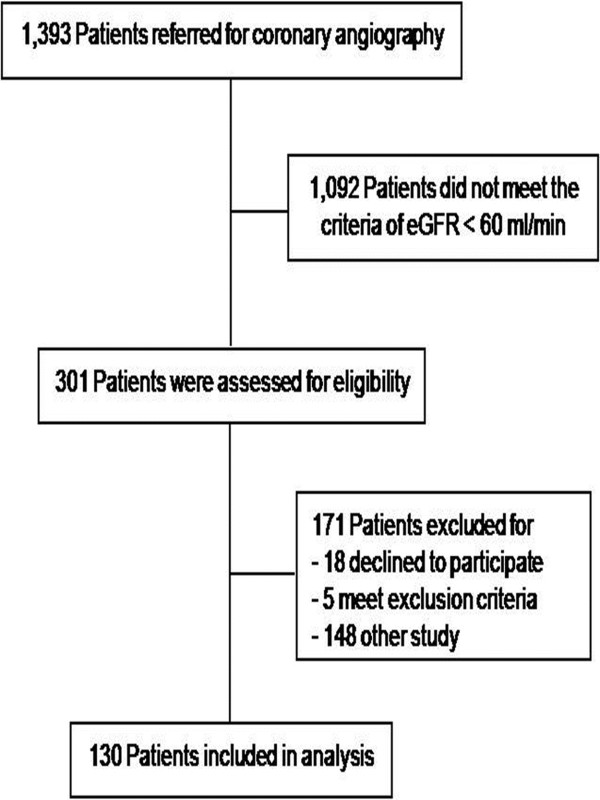
Diagram showing the flow of patients through each step of trial.

A total of 1,393 patients referred for coronary angiography and/or intervention were screened between January 2010 and December 2011 (Figure 1). Of these patients, 301 patients met the inclusion criteria based on a baseline eGFR < 60 mL/min. 171 patients were excluded from the study because 18 patients declined to participate, two patients suffered from congestive heart failure, three patients developed AKI and 148 patients were enrolled in other study. Finally, 130 CKD patients were included in the present study: CKD stage III and CKD stage IV numbered 100 and 30 patients, respectively.

After coronary procedures, 16/130 (12.31%) patients developed CI-AKI; 13 patients for CI-AKI stage I, and 3 patients for CI-AKI stage II. CI-AKI developed in 10/100 (10%) in patients with CKD stage III; 9 patients for CI-AKI stage I, and 1 patient for CI-AKI stage II. While, CI-AKI developed in 6/30 (20%) in patients with CKD stage IV; 4 patients for CI-AKI stage I, and 2 patients for CI-AKI stage II. Based on the primary outcome, we classified subjects into those with and without CI-AKI (No CI-AKI).

The baseline clinical characteristics of patients, stratified by the presence/absence of baseline CI-AKI are shown in Table 1. Comparing these two groups, there were no statistically significant differences in age, sex, blood pressure, heart rate, body mass index, concomitant medication, and co-morbidity, including hypertension, diabetes mellitus, hypercholesterolemia, left ventricular ejection fraction, myocardial infarction, Canadian Cardiovascular Society classification, New York Heart Association classification, angiography/percutaneous intervention and number of vessel disease. Similarly, no statistically significant differences between the groups, especially contrast volume, intravenous volume and risk score.

**Table 1:** /Baseline demographics in patients undergoing elective coronary procedures

**Characteristic**	**No CI-AKI**	**CI-AKI**	**CI-AKI (S1)**	**CI-AKI (S2)**	**P value**
Number of subject	114	16	13	3	
CKD stage III: CKD stage IV	90:24	10:6	9:4	1:2	
Age (yr)	70 ± 10	72 ± 7	73 ± 5	70 ± 9	0.67
Men: women	88:26	12:4	10:3	2:1	0.17
SBP (mmHg)	129 ± 17	125 ± 13	125 ± 14	124 ± 14	0.65
DBP(mmHg)	71 ± 10	69 ± 12	70 ± 12	67 ± 8	0.74
Heart rate	75 ± 10	74 ± 12	76 ± 11	72 ± 8	0.48
Height (cm)	162 ± 9	161 ± 9	160 ± 7	162 ± 8	0.85
Weight (kg)	60 ± 13	56 ± 7	58 ± 5	54 ± 8	0.16
Body mass index (kg/m2)	24 ± 4	23 ± 3	23 ± 3	21 ± 4	0.14
Diabetes mellitus (%)	42	44	46	33	0.25
Hypertension (%)	80	75	77	67	0.19
Hypercholesterolemia (%)	50	44	38	67	0.16
Myocardial infarction (%)	18	25	23	33	0.37
LVEF < 40% (%)	20	19	15	33	0.59
CCS > II (%)	96	94	92	100	0.49
NYHA > II (%)	44	44	38	67	0.6
Angiogram/PCI (%)	66	75	85	67	0.58
Number of vessel disease > 1 (%)	61	63	50	67	0.62
ACEI (%)	30	31	31	33	0.42
ATRA (%)	40	31	31	33	0.48
Beta blocker (%)	54	44	38	67	0.56
CCB (%)	28	31	31	33	0.26
Diuretic (%)	26	31	31	33	0.64
Aspirin (%)	72	63	62	67	0.51
Statin (%)	48	44	38	67	0.17
Nitrate (%)	38	38	31	67	0.14
Contrast volume (ml)	126 ± 65	129 ± 90	126 ± 80	124 ± 92	0.43
IV volume (ml)	1483 ± 453	1591 ± 260	1598 ± 246	1562 ± 128	0.48
Total risk score	8 ± 3	8 ± 3	8 ± 3	9 ± 1	0.24

CI-AKI indicates contrast induced acute kidney injury, CI-AKI (S1) indicates CI-AKI stage I, CI-AKI (S2) indicates CI-AKI stage II, SBP indicates systolic blood pressure, DBP indicates diastolic blood pressure, LVEF indicates left ventricular ejection fraction, CCS indicates Canadian Cardiovascular Society, NYHA indicates New York Heart Association, PCI indicates percutaneous intervention, CCB indicates calcium channel blocker.

The baseline, follow-up and absolute changes (from baseline) for SCr, eGFR, uNGAL and delta of uNGAL in each group of all patients, CKD stage III and CKD stage IV are summarized in Table 2. In all patients and CKD stage IV patients (but not CKD stage III patients), the CI-AKI group had a significantly higher SCr but lower eGFR at baseline (P < 0.05) (Table 2A, 2B and 2C). The CI-AKI group had a significant increase in SCr and decrease in eGFR at 48 h post coronary procedures for all patients, CKD stage III and IV patients (P < 0.05). On average at 48 h, patients in the CI-AKI group had a statistically significant increase in SCr of 55% (all patients), 73% (CKD stage III) and 42% (CKD stage IV), while corresponding decrease in eGFR were 33.33%, 50% and 35% (P < 0.05) respectively. The mean SCr and eGFR did not change significantly in the No CI-AKI group (Table 2).

**Table 2:** Baseline, follow-up and absolute changes (from baseline) for serum creatinine, eGFR, urine NGAL and delta of urine NGAL in each group of all patients (2A), CKD stage III (2B) and CKD stage IV (2C)

**2A**				
**Characteristic**	**All patients**			
	**No CI-AKI**	**CI-AKI**	**CI-AKI (S1)**	**CI-AKI (S2)**
	**( N = 114)**	**(N = 16)**	**(N = 13)**	**(N = 3)**
Serum creatinine (mg/dL)				
Baseline	1.4 ± 0.4	2.0 ± 0.6^a^	2.0 ± 0.6^a^	1.9 ± 0.3^a^
Follow-up (48 hr after)	1.3 ± 0.4	3.1 ± 1.1^a^	2.7 ± 1.1^a^	4.5 ± 0.4^a,b^
Absolute change	0.0 ± 0.2	1.1 ± 0.9^a^	0.7 ± 0.9^a^	2.7 ± 0.6^a,b^
eGFR (ml/min)				
Baseline	44 ± 16	27 ± 7^a^	27 ± 8^a^	28 ± 3^a^
Follow-up (48 hr after)	45 ± 17	18 ± 6^a^	20 ± 6^a^	11 ± 2^a,b^
Absolute change	1 ± 5	-9 ± 5^a^	-7 ± 3^a^	-17 ± 2^a,b^
Urine NGAL (ng/ml)				
Baseline	68 ± 99	144 ± 111^a^	154 ± 143^a^	113 ± 45^a^
3 h	79 ± 102	211 ± 120^a^	213 ± 159^a^	202 ± 60^a^
6 h	93 ± 129	267 ± 153^a^	255 ± 191^a^	316 ± 86^a^
12 h	115 ± 166	277 ± 169^a^	256 ± 181^a^	352 ± 133^a^
18 h	132 ± 175	298 ± 171^a^	315 ± 187^a^	272 ± 80^a^
24 h	122 ± 165	294 ± 162^a^	314 ± 173^a^	265 ± 52^a^
Delta urine NGAL (ng/ml)				
0-3 h	10 ± 92	66 ± 78^a^	59 ± 81^a^	99 ± 61^a^
0-6 h	24 ± 110	122 ± 87^a^	101 ± 72^a^	213 ± 101^a,b^
0-12 h	42 ± 158	132 ± 91^a^	112 ± 76^a^	242 ± 104^a,b^
0-18 h	59 ± 142	135 ± 136^a^	137 ± 147^a^	159 ± 95^a^
0-24 h	49 ± 149	149 ± 136^a^	160 ± 136^a^	151 ± 80^a^
**2B**				
**Characteristic**	**CKD stage III**			
	**No CI-AKI)**	**CI-AKI**	**CI-AKI (S1)**	**CI-AKI (S2)**
	**(N = 90**	**(N = 10)**	**(N = 9)**	**(N = 1)**
Serum creatinine (mg/dL)				
Baseline	1.3 ± 0.3	1.5 ± 0.5	1.5 ± 0.7	1.6
Follow-up (48 hr after)	1.2 ± 0.3	2.7 ± 1.0^a^	2.5 ± 0.9^a^	4.6^a,b^
Absolute change	-0.1 ± 0.2	1.1 ± 0.8^a^	0.9 ± 0.3^a^	3.0^a,b^
eGFR (ml/min)				
Baseline	46 ± 14	44 ± 9	43 ± 9	43
Follow-up (48 hr after)	48 ± 11	22 ± 8^a^	24 ± 8^a^	14^a,b^
Absolute change	1 ± 6	-22 ± 9^a^	-18 ± 6^a^	-29^a,b^
Urine NGAL (ng/ml)				
Baseline	61 ± 81	72 ± 45	75 ± 42	98
3 h	75 ± 83	145 ± 73^a^	136 ± 72^a^	220^a^
6 h	84 ± 119	171 ± 75^a^	160 ± 70^a^	264^a^
12 h	113 ± 162	181 ± 86^a^	178 ± 78^a^	208^a^
18 h	121 ± 161	205 ± 210^a^	203 ± 79^a^	226^a^
24 h	114 ± 163	210 ± 87^a^	210 ± 93^a^	204^a^
Delta of urine NGAL (ng/ml)				
0-3 h	13 ± 80	63 ± 42^a^	62 ± 64^a^	122^a^
0-6 h	22 ± 104	89 ± 87^a^	85 ± 86^a^	166^a^
0-12 h	49 ± 136	99 ± 91^a^	103 ± 63^a^	110^a^
0-18 h	54 ± 131	124 ± 136^a^	128 ± 55^a^	128^a^
0-24 h	46 ± 143	128 ± 136^a^	135 ± 82^a^	106^a^
**2C**				
**Characteristic**	**CKD stage IV**			
	**No CI-AKI**	**CI-AKI**	**CI-AKI (S1)**	**CI-AKI (S2)**
	**(N = 24)**	**(N = 6)**	**(N = 4)**	**(N = 2)**
Serum creatinine (mg/dL)				
Baseline	1.7 ± 0.5	2.4 ± 0.6^a^	2.3 ± 0.6^a^	2.5 ± 0.3^a^
Follow-up (48 hr after)	1.6 ± 0.6	3.3 ± 1.3^a^	2.9 ± 0.9^a^	4.5 ± 0.6^a,b^
Absolute change	-0.1 ± 0.3	1.0 ± 1.2^a^	0.6 ± 0.4^a^	2.1 ± 0.8^a,b^
eGFR (ml/min)				
Baseline	27 ± 3	20 ± 4^a^	21 ± 5^a^	19 ± 3^a^
Follow-up (48 hr after)	27 ± 4	14 ± 6^a^	15 ± 8^a^	9 ± 4^a,b^
Absolute change	0 ± 3	-7 ± 6^a^	-6 ± 4^a^	-10 ± 4^a,b^
Urine NGAL (ng/ml)				
Baseline	102 ± 98	217 ± 180^a^	216 ± 231^a^	218 ± 145^a^
3 h	116 ± 157	314 ± 251^a^	340 ± 318^a^	261 ± 153^a^
6 h	134 ± 162	404 ± 227^a^	444 ± 280^a^	324 ± 124^a^
12 h	132 ± 191	409 ± 192^a^	415 ± 239^a^	396 ± 107^a^
18 h	193 ± 230	394 ± 221^a^	348 ± 226^a^	349 ± 169^a^
24 h	164 ± 182	385 ± 216^a^	362 ± 131^a^	329 ± 148^a^
Delta of urine NGAL (ng/ml)				
0-3 h	10 ± 156	97 ± 92^a^	124 ± 106^a^	84 ± 138^a^
0-6 h	33 ± 131	187 ± 103^a^	227 ± 101^a^	217 ± 159^a^
0-12 h	20 ± 227	192 ± 108^a^	198 ± 108^a^	248 ± 152^a^
0-18 h	62 ± 186	112 ± 208^a^	132 ± 112^a^	162 ± 183^a,^
0-24 h	63 ± 174	168 ± 212^a^	145 ± 110^a^	212 ± 192^a,^

eGFR indicates estimated glomerular filtration rate.

^a^*P* <0.05 compared with No-CI-AKI.

^b^*P* <0.05 compared with CI-AKI (S1).

In all patients and patients with CKD stage IV, the mean uNGAL levels at baseline were significantly higher in the CI-AKI when compared to No CI-AKI group (P < 0.05) (Table 2A and 2C) but without a statistically significant difference in CKD stage III patients (Table 2B). The CI-AKI group had a significant increase in uNGAL and delta of uNGAL at all time points post coronary procedures when compared to No CI-AKI group (P < 0.05). Comparing CI-AKI stages I and II, there were not significant differences in uNGAL levels at any time point post coronary procedures. However, delta of uNGAL levels at 0–6 h and 0–12 h were significantly higher in the CI-AKI stage II than the CI-AKI stage I (P < 0.05) (Table 2A) respectively.

**Figure 2:**
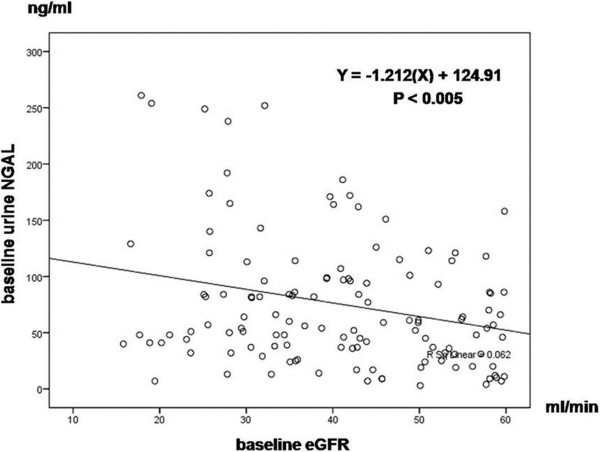
Univariate baseline statistical correlations (Pearson coefficient) of urine NGAL and baseline eGFR.

The uNGAL was significantly and inversely correlated with eGFR (*R =* 0.25, P < 0.005; Figure 2), and hemoglobin (*R* = 0.44, P < 0.01). However, uNGAL did not correlate with other parameters, such as SCr, age, gender, blood pressure, height, body weight and body mass index (P > 0.05).

**Figure 3:**
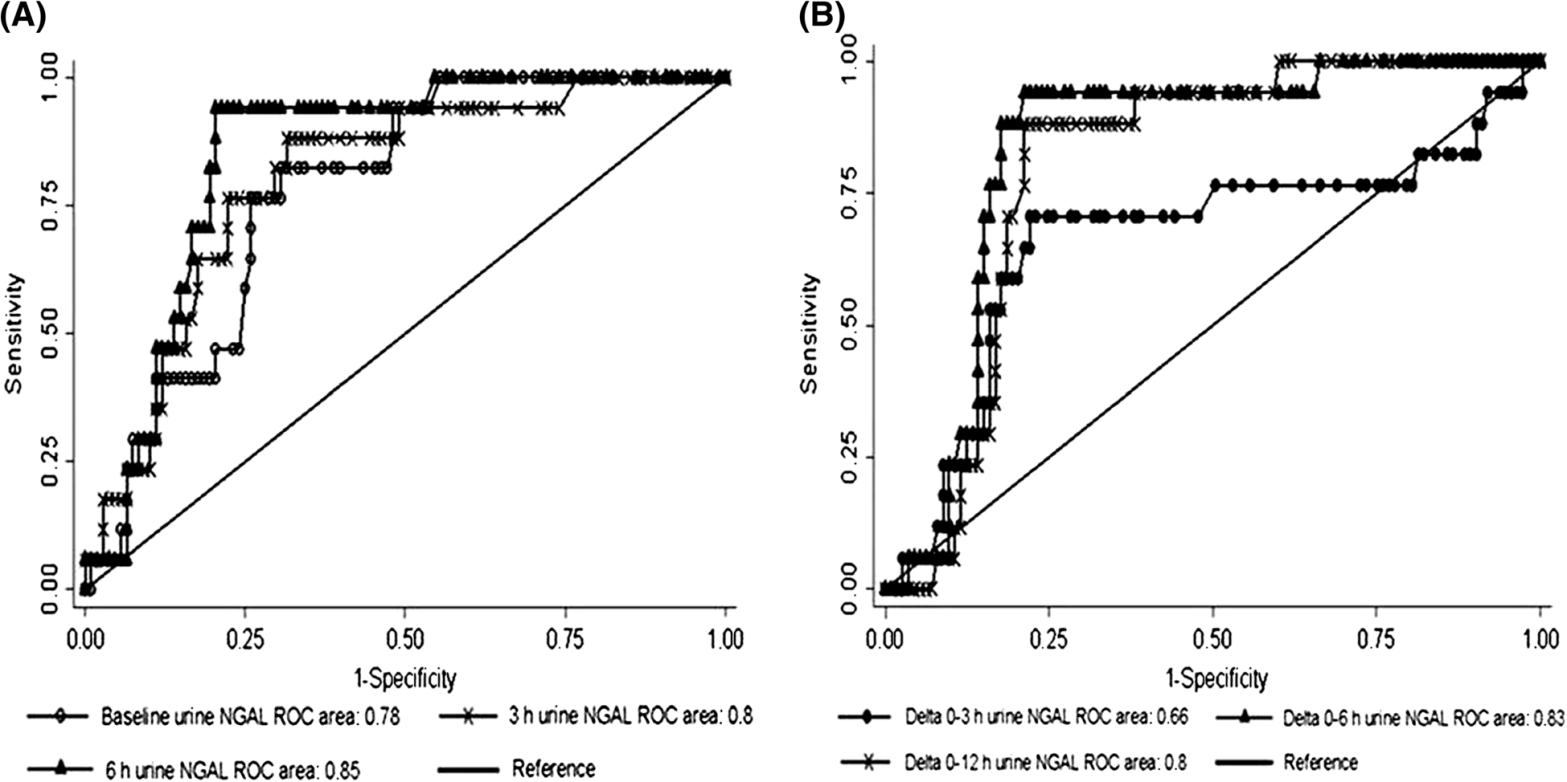
Receiver operating characteristics (ROC) curves of urine NGAL at baseline, 3 h, and 6 h (3A) and delta of urine NGAL 0–3 h, 0–6 h, and 0–12 h (3B) considering incidence of CI-AKI as status variable.

The performance characteristics of uNGAL and delta of uNGAL for diagnosis CI-AKI are shown in Table 3. The ROC curves AUCs ranged from 0.78 to 0.86 (uNGAL), and 0.66 to 0.83 (delta of uNGAL) (Table 3A). The highest AUCs for uNGAL and delta of uNGAL for diagnosis CI-AKI were 0.85 (95% CI = 0.76–0.91; P < 0.01) at 6 h (Figure 3A) and 0.83 (95% CI = 0.75–0.92; P < 0.01) at 0–6 h (Figure 3B), respectively. The cut-off value of uNGAL for optimal combination of sensitivity (94%) and specificity (78%) was 117 ng/mL, while the cut-off value of delta of uNGAL for optimal combination of sensitivity (88%) and specificity (80%) was 45 ng/mL, respectively. The cut-off value of uNGAL and delta of uNGAL for diagnosis CI-AKI in CKD stage III were similar to all patients (Table 3B). However, the cut-off value of uNGAL and delta of uNGAL for diagnosis CI-AKI in CKD stage IV were higher than CKD stage III and all patients (Table 3B).

**Table 3:** Diagnostic characteristics of urine NGAL for CI-AKI, stratified by baseline and postoperative time in all patient (3A), CKD stage III and CKD stage IV (3B)

**3A**								
**All patients (N = 130)**								
	**ROC (95% CI)**	**Cut point (ng/ml)**	**Sensitivity (%)**	**Specificity (%)**				
uNGAL								
baseline	0.78 (0.68 to 0.87)	75	77	74				
3 h	0.80 (0.69 to 0.89)	104	77	76				
6 h	0.85 (0.76 to 0.91)	117	94	78				
12 h	0.82 (0.74 to 0.90)	142	88	76				
18 h	0.84 (0.76 to 0.91)	161	82	76				
24 h	0.86 (0.79 to 0.93)	184	82	81				
Delta of uNGAL								
0-3 h	0.66 (0.49 to 0.79)	22	85	78				
0-6 h	0.83 (0.75 to 0.92)	45	88	80				
0-12 h	0.80 (0.72 to 0.89)	71	88	79				
0-18 h	0.78 (0.67 to 0.90)	77	82	73				
0-24 h	0.79 (0.67 to 0.91)	71	77	74				
**3B**								
	**CKD stage III (N = 100)**				**CKD stage IV (N = 30)**			
	**ROC (95% CI)**	**Cut point (ng/ml)**	**Sensitivity (%)**	**Specificity (%)**	**ROC (95% CI)**	**Cut point (ng/ml)**	**Sensitivity (%)**	**Specificity (%)**
uNGAL								
baseline	0.72 (0.61 to 0.83)	61	70	71	0.87 (0.73 to 1.0)	84	86	73
3 h	0.79 (0.65 to 0.93)	108	80	78	0.88 (0.75 to 1.0)	155	86	82
6 h	0.82 (0.72 to 0.92)	117	92	80	0.89 (0.80 to 1.0)	207	86	86
12 h	0.77 (0.67 to 0.88)	142	84	75	0.86 (0.72 to 0.99)	281	86	83
18 h	0.81 (0.72 to 0.89)	161	70	76	0.83 (0.68 to 0.97)	285	83	78
24 h	0.82 (0.74 to 0.92)	184	78	81	0.88 (0.75 to 1.0)	235	86	83
Delta of uNGAL								
0-3 h	0.80 (0.64 to 0.96)	24	90	78	0.75 (0.49 to 1.0)	22	86	78
0-6 h	0.82 (0.69 to 0.95)	42	91	79	0.89 (0.77 to 1.0)	54	100	87
0-12 h	0.76 (0.64 to 0.88)	65	82	77	0.88 (0.75 to 1.0)	71	100	83
0-18 h	0.80 (0.71 to 0.89)	77	88	73	0.73 (0.47 to 1.0)	102	86	83
0-24 h	0.81 (0.70 to 0.91)	61	80	74	0.73 (0.47 to 0.99)	91	71	78

The performance characteristics of uNGAL and delta of uNGAL for diagnosis CI-AKI stage I are shown in Table 4. The best AUCs for uNGAL and delta of uNGAL for diagnosis CI-AKI stage I were 0.83 (95% CI = 0.74–0.92; P <0.01) at 6 h and 0.82 (95% CI = 0.72–0.92; P < 0.01) at 0–6 h post coronary procedure (Table 4A). The sensitivities were 92% and 85% while the specificities were 76% and 80% when the cut-off values of uNGAL and delta of uNGAL were 117 and 54 ng/mL (Table 4A), respectively. The cut-off value of uNGAL and delta of uNGAL for diagnosis CI-AKI stage I in CKD stage III were similar to all patients (Table 4B). However, the cut-off value of uNGAL and delta of uNGAL for diagnosis CI-AKI stage I in CKD stage IV were higher than CKD stage III and all patients (Table 4B).

**Table 4:** Diagnostic characteristics of urine NGAL for CI-AKI stage I, stratified by baseline and postoperative time in all patient (4A), CKD stage III and CKD stage IV (4B)

**4A**								
**All Patients (N = 130)**								
	**ROC (95% CI)**	**Cut point (ng/ml)**	**Sensitivity (%)**	**Specificity (%)**				
uNGAL								
baseline	0.77 (0.65 to 0.89)	74	77	72				
3 h	0.82 (0.71 to 0.94)	108	85	76				
6 h	0.83 (0.74 to 0.92)	117	92	76				
12 h	0.82(0.72 to 0.91)	142	85	73				
18 h	0.84 (0.76 to 0.92)	161	83	74				
24 h	0.85 (0.77 to 0.93)	181	85	79				
Delta of uNGAL								
0-3 h	0.75 (0.58 to 0.91)	22	85	78				
0-6 h	0.82 (0.72 to 0.92)	54	85	80				
0-12 h	0.79 (0.68 to 0.88)	71	85	76				
0-18 h	0.77 (0.63 to 0.91)	77	92	72				
0-24 h	0.77 (0.62 to 0.92)	71	77	73				
**4B**								
	**CKD stage III (N = 100)**				**CKD stage IV (N = 30)**			
	**ROC (95% CI)**	**Cut point (ng/ml)**	**Sensitivity (%)**	**Specificity (%)**	**ROC (95% CI)**	**Cut point (ng/ml)**	**Sensitivity (%)**	**Specificity (%)**
uNGAL								
baseline	0.70 (0.58 - 0.81)	41	78	58	0.90 (0.78 - 1.0)	140	100	88
3 h	0.77 (0.62 - 0.92)	108	78	78	0.89 (0.77 - 1.0)	162	100	79
6 h	0.80 (0.70 - 0.91)	117	89	79	0.89 (0.75 - 1.0)	239	100	80
12 h	0.76 (0.69 - 0.87)	142	78	74	0.83 (0.66 1.0)	276	100	63
18 h	0.80 (0.70 - 0.89)	130	78	72	0.85 (0.68 - 1.0)	242	100	73
24 h	0.82 (0.72 - 0.92)	160	78	79	0.90 (0.78 - 1.0)	184	100	77
Delta of uNGAL								
0-3 h	0.79 (0.62 - 0.96)	24	89	77	0.81 (0.64 - 0.97)	22	100	73
0-6 h	0.80 (0.66 - 0.94)	42	89	78	0.84 (0.70 - 0.98)	65	100	77
0-12 h	0.75 (0.63 - 0.89)	74	78	77	0.82 (0.66 - 0.97)	71	100	76
0-18 h	0.81 (0.71 - 0.90)	77	89	73	0.61 (0.19 - 1.0)	102	75	73
0-24 h	0.81 (0.70 - 0.90)	71	78	77	0.62 (0.19 - 1.0)	44	75	54

The performance characteristics of uNGAL and delta of uNGAL for diagnosis CI-AKI stage II are shown in Table 5. The best AUCs for uNGAL and delta of uNGAL for diagnosis CI-AKI stage II were 0.9 (95% CI = 0.84–0.97; P < 0.01) at 6 h and 0.93 (95% CI = 0.86–0.99; P < 0.01) at 0–6 h post coronary procedure (Table 5A). The cut-off value of uNGAL for optimal combination of sensitivity (100%) and specificity (87%) was 264 ng/mL, while the cut-off value of delta of uNGAL for optimal combination of sensitivity (100%) and specificity (87%) was 121 ng/mL (Table 5A). The cut-off value of uNGAL and delta of uNGAL for diagnosis CI-AKI stage II in CKD stage III were similar to all patients (Table 5B). In addition, the cut-off value of uNGAL and delta of uNGAL for diagnosis CI-AKI stage II in CKD stage IV were higher than CKD stage III and all patients (Table 5B).

**Table 5:** Diagnostic characteristics of urine NGAL for CI-AKI stage II, stratified by baseline and postoperative time in all patient (5A), CKD stage III and CKD stage IV (5B)

**5A**								
**All patients (N = 130)**								
	**ROC (95% CI)**	**Cut point (ng/ml)**	**Sensitivity (%)**	**Specificity (%)**				
uNGAL								
baseline	0.76 (0.65 to 0.88)	82	100	70				
3 h	0.86 (0.76 to 0.97)	135	100	77				
6 h	0.9 (0.84 to 0.97)	264	100	87				
12 h	0.85 (0.74 to 0.96)	204	100	79				
18 h	0.8 (0.72 to 0.88)	186	100	76				
24 h	0.78 (0.7 to 0.86)	175	100	74				
Delta of uNGAL								
0-3 h	0.87 (0.77 to 0.97)	53	100	81				
0-6 h	0.93 (0.86 to 0.99)	121	100	87				
0-12 h	0.82 (0.67 to 0.98)	65	100	71				
0-18 h	0.77 (0.63 to 0.91)	83	100	67				
0-24 h	0.76 (0.64 to 0.88)	61	100	68				
**5B**								
	**CKD stage III (N = 100)**				**CKD stage IV (N = 30)**			
	**ROC (95% CI)**	**Cut point (ng/ml)**	**Sensitivity (%)**	**Specificity (%)**	**ROC (95% CI)**	**Cut point (ng/ml)**	**Sensitivity (%)**	**Specificity (%)**
uNGAL								
baseline	0.88 (0.0 to 1.0)	143	100	88	0.60 (0.42 to 0.79)	82	100	59
3 h	0.92 (0.0 to 1.0)	220	100	92	0.74 (0.48 to 1.0)	135	100	63
6 h	0.93 (0.0 to 1.0)	264	100	93	0.80 (0.56 to 1.0)	281	100	71
12 h	0.83 (0.0 to 1.0)	208	100	83	0.77 (0.51 to 1.0)	204	100	65
18 h	0.83 (0.0 to 1.0)	226	100	83	0.67 (0.48 to 0.85)	186	100	63
24 h	0.81 (0.0 to 1.0)	204	100	81	0.70 (0.52 to 0.87)	175	100	68
Delta of uNGAL								
0-3 h	0.84 (0.0 to 1.0)	77	100	84	0.88 (0.72 to 1.0)	53	100	82
0-6 h	0.90 (0.0 to 1.0)	121	100	90	0.91 (0.77 to 1.0)	197	100	86
0-12 h	0.72 (0.0 to 1.0)	65	100	72	0.88 (0.68 to 1.0)	120	100	78
0-18 h	0.69 (0.0 to 1.0)	83	100	69	0.78 (0.61 to 0.95)	102	100	71
0-24 h	0.70 (0.0 to 1.0)	61	100	70	0.79 (0.60 to 0.98)	91	100	71

## Discussion

This prospective study in CKD patients undergoing coronary procedure has demonstrated that uNGAL at 6 h and delta of uNGAL 0–6 h post coronary procedure were good biomarkers for early diagnosis CI-AKI and had some value in grading its severity. Baseline uNGAL inversely correlated with the eGFR but did not correlate with the SCr, suggesting it is a better marker for renal impairment than SCr. Thus, monitoring the baseline uNGAL could be a simple surrogate marker correspond to baseline renal function and following the uNGAL especially at 6 h after coronary procedure predict the development and severity of CI-AKI.

The findings from the present study confirm that uNGAL is an important biomarker reflects the residual renal function in CKD patients similar to previous clinical studies have been reported [33-35] which explain the role of this protein in glomerular filtration and tubular adaptation during chronic kidney injury. NGAL was originally expressed in activated human neutrophils, but it is also expressed at low levels in different human tissues, including the distal nephron of kidney [36]. NGAL is synthesized systemically during kidney injury followed by glomerular filtration and impaired proximal tubular reabsorption. Moreover, NGAL was produced locally by injured distal nephron epithelium resulting in elevated of uNGAL [16]. Hence, sustained production of NGAL by the chronic stressed kidney is responsible for the increase uNGAL levels that reflect the residual renal function superior than SCr in CKD patients.

Serial uNGAL in our CKD patients rose early since 3 h and peaked at 18 h after coronary procedures. These kinetics are quite different to the patients with normal renal function [26,28] whose uNGAL concentrations peak earlier at 6–8 h and begin to return at 8–12 h after coronary procedures. This difference is probably due to the delayed excretion of contrast media in CKD patients with continuing contrast induced kidney injury. Not surprisingly, our baseline and serial uNGAL concentrations are higher than those reported from studies with patients with normal renal function.

Previous studies have shown the value of uNGAL for early diagnosis CI-AKI in patients with normal renal function undergoing coronary procedures [25-27]. Hirsch et al. [25] determined the diagnosis of CI-AKI in 91 congenital heart disease pediatric patients with normal renal function undergoing cardiac catheterization and angiography. The mean baseline uNGAL level was 17.7 ng/mL and the levels of uNGAL increased 7 fold in CI-AKI patients at 2 h after procedures. Using a cut-off value of uNGAL 100 ng/ml at 6 h, the sensitivity, specificity, and AUC for predicting CI-AKI were excellent; 90%, 99%, and 0.97, respectively. Ling et al. [27] reported the diagnosis of CI-AKI in adult patients with normal renal function undergoing coronary angiography. Baseline uNGAL level was 5.76 ng/mL and increased two fold in CI-AKI patients at 24 h after procedures. Using a cut-off value of 9.85 ng/mL, the sensitivity, specificity, and AUC for prediction of CI-AKI were good for the 24 h uNGAL (77%, 70%, and 0.73, respectively). Bachorzewska-Gajewska et al. [26] determined the diagnosis of CI-AKI in 100 patients with normal SCr undergoing coronary interventions. The mean baseline uNGAL level was 9.9 ng/mL and this increased significantly two fold at 4 h after interventions. Furthermore, the uNGAL levels were significantly higher in the patients with CI-AKI at 4 h after procedures when compared with patients without CI-AKI. Broadly, these studies had the baseline and follow-up uNGAL levels after coronary procedures lower than the present study because the population in the present study was CKD patients who had renal dysfunction which delay the kinetic change of uNGAL. Thus, the cut-off points of uNGAL for detecting CI-AKI in CKD patients were higher than patients with normal renal function.

In the present study, uNGAL and delta of uNGAL were excellent biomarkers for detecting the development CI-AKI in CKD patients undergoing elective coronary procedures. The best time was 6 h for uNGAL and between 0 to 6 h for delta of uNGAL. Therefore, at least 2 time points post baseline are needed for measuring uNGAL and calculating delta of uNGAL to detect the development of CI-AKI. More intense sampling may have shown an earlier time to detect CI-AKI. At these times, the uNGAL and delta of uNGAL levels were higher in the CKD stage IV than the CKD stage III patients but numbers are small and concern is advised in generalizing these results. Caution should be exercised when comparing across studies because studies have used different patients population, diagnostic criteria of CI-AKI definitions, storage conditions of urine specimens, and techniques for measuring uNGAL and others confounding factors.

Cut-off points of uNGAL at 6 h and delta of uNGAL 0–6 h predict the severity of CI-AKI in CKD patients undergoing elective coronary procedures. Although the numbers of CI-AKI patients in the present study were small but there was a suggestion that uNGAL and delta of uNGAL levels were higher on patients who developed CI-AKI stage II when compared to those with CI-AKI stage I for 2–3 times. This is an area where more data are needed to validate the levels of uNGAL, the degree of renal impairment and if this also predicts later morbidity.

The present study has limitations. First, the present study has only been conducted in a single center. These results will need to be validated in a larger population by perform a multi-center study. Second, NGAL measurement by ELISA kit in the present study detect the high molecular weight NGAL complex which may lack sensitivity. Nickolas et al. [37] have demonstrated that specific measurement of monomeric NGAL was correlated with GFR and renal cell types. Thus, a more specific assay of the NGAL monomer could be developed for early detect CI-AKI. Finally, several potential urine and plasma proteins were demonstrated to early diagnosis CI-AKI. It could take the advantage if the author will combine two or more biomarkers at different time points from 0–12 h after coronary procedure which can improve the diagnostic accuracy.

## Conclusions

Monitoring of uNGAL levels at baseline and 6 h post elective coronary procedures in CKD patients had high sensitive and specificity for early diagnosis CI-AKI and showed some discriminatory value for CI-AKI severity. It should be used in favour of SCr for identifying early patients who develop CI-AKI.

## Abbreviations

(CI-AKI): Contrast-induced acute kidney injury; (CKD): Chronic kidney disease; (SCr): Serum creatinine; (NGAL): Neutrophil gelatinase-associated lipocalin; (eGFR): Estimation of glomerular filtration rate; (ROC) curves: Receiver-operating characteristic; (AUC): Area under the curve; (u): Urine.

## Competing interests

All authors declare that they have no competing interest.

## Authors’ contributions

AT designed the protocol, collected the data, NGAL ELISA kit analysis, analyzed the results and wrote the manuscript. PH and DP collected the data, recruiting the patients and revised the manuscript. All authors read and approved the final manuscript.

## Pre-publication history

The pre-publication history for this paper can be accessed here:

http://www.biomedcentral.com/1471-2369/14/270/prepub
